# Desialylation of Atg5 by sialidase (Neu2) enhances autophagosome formation to induce anchorage-dependent cell death in ovarian cancer cells

**DOI:** 10.1038/s41420-020-00391-y

**Published:** 2021-02-01

**Authors:** Eswara Murali Satyavarapu, Shalini Nath, Chitra Mandal

**Affiliations:** Cancer Biology and Inflammatory Disorder Division, Council of Scientific and Industrial Research-Indian Institute of Chemical Biology, 4, Raja S.C. Mallick Road, Kolkata, 700032 India

**Keywords:** Ovarian cancer, Apoptosis

## Abstract

Increased sialylation is one of the hallmarks of ovarian cancer (OC) but its relation with programmed cell death is not known. Here we explored the molecular interplay between autophagy, apoptosis/anoikis, and aberrant-expression of the PI3K-Akt/mTOR pathway in the context of sialidase. OC is accompanied by low expression of cytosolic sialidase (Neu2) and ~10-fold more α2,6- than α2,3-linked sialic acids found through qPCR, western blot, and flow cytometry. Interestingly, Neu2 overexpression cleaved α2,6- and α2,3-linked sialic acids and reduced cell viability. Several autophagy-related molecules like LC3B/Atg3/Atg5/Atg7/Atg12/Atg16L1/Beclin1 were upregulated upon Neu2 overexpression. Atg5, a crucial protein for autophagosome formation, was desialylated by overexpressed Neu2. Desialylated Atg5 now showed enhanced association both with Atg12 and Atg16L1 leading to more autophagosome formation. Neu2-overexpressing cells exhibited extrinsic pathway-mediated apoptosis as reflected the in activation of Fas/FasL/FADD/Bid/caspase 8/caspase 6/caspase 3/PARP cleavage. There was also increased Bax, reduced Bcl2, and several cell-cycle molecules (CDK2/CDK4/CDK6/cyclin-B1/cyclin-E). Inhibition of autophagy using bafilomycin A1 or Beclin1 siRNA leads to reversal of Neu2-induced apoptosis suggesting their possible relationship. Additionally, overexpressed Neu2 inhibited growth factor-mediated signaling molecules involved in the PI3K/Akt-mTOR pathway probably through their desialylation. Furthermore, overexpressed Neu2 inhibited epithelial (ZO-1/Claudin1), mesenchymal (snail/slug), and cell-adhesion (integrin-β3/focal-adhesion kinase) molecules suggesting anchorage-dependent cell death (anoikis). Such changes were absent in the presence of bafilomycin A1 indicating the involvement of autophagy in Neu2-induced anoikis. The physiological relevance of our in vitro observations was further confirmed in the OC xenograft model. Taken together, it is the first report demonstrating that Atg5 is a sialoglycoprotein having α2,6- and α2,3-linked sialic acids and its desialylation by overexpressed Neu2 leads to its activation for autophagosome formation, which induced apoptosis/anoikis in OC.

## Introduction

Ovarian cancer (OC) causes 152,000 deaths worldwide annually due to late diagnosis and prognosis^1^. Higher sialylation is a common feature in cancers that are modulated mainly by sialyltransferases and sialidases^2^. Hence, alteration of these enzymes affects various signaling pathways, thus promoting tumor growth^3^. Increased cancer cell surface sialylation^4^, elevated levels of sialic acids (SAs) in the OC patient’s serum^5^, and higher expression of sialyltransferases (ST3GAL1/ST6GAL1) are possibly responsible for chemoresistance and relapse^6–8^.

Mammalian sialidases are enzymes that cleave the linkage-specific SA from sialoglycoproteins and are classified as lysosomal (Neu1), cytosolic (Neu2), membrane bound (Neu3), and luminal (Neu4)^9^. The role of these sialidases in different cancer varies differently. Overexpression of Neu2 helps cell survival in prostate cancer, where it leads to apoptosis in leukemia^10^ and pancreatic cancer stem-like cells^11^, suggesting a context-specific role of this enzyme. Here we aimed to decipher the role of Neu2 in OC.

Autophagy is a self-eating process where damaged/inactive cytoplasmic proteins and cellular organelles are initially enveloped in a double-membrane vesicle called autophagosomes followed by fusion with lysosomes. Subsequently, all the contents in the autophagosomes are degraded by lysosomal hydrolyses leading to the generation of energy. This process is supported by different autophagy-related molecules called Atg proteins, namely, Atg 3, 5, 7, 12, 16L1, and Beclin1. Autophagosome formation essentially requires LC3B-II and Atg5-12-16L1 complex, important markers of autophagy. LC3B-II is formed through conjugation of LC3B-I with phosphatidyl-ethanolamine and is recruited to autophagosomal membranes^12^. We have earlier demonstrated the autophagy-independent role of LC3B in inducing apoptosis of OC cells^13^. Accordingly, mechanisms involved in autophagy-induced cell death needs further study.

Autophagy generally helps in cell survival during starved conditions^14^. However, hyper-enhancement of autophagy due to chemotherapy or any genetic abnormalities activates apoptosis machinery leading to apoptosis in cancer^15^. Therefore, both autophagy inhibition and hyper-induction lead to cell death. Thus it will be interesting to understand how sialidases can modulate autophagy that might help us to explore the molecular relationship between important autophagy-related molecules and sialidase.

Aberrant expression of the phosphoinositide-3 kinase (PI3K)-Akt/mammalian target of rapamycin (mTOR) pathway is reported in cancers^16^. In OC, constitutive activation of this pathway enhances its metastatic potential and chemoresistance^17^. This pathway directs autophagy in cancer cells^18^. Sialylation controls the activity of many molecules involved in this pathway^19–21^; hence, it is important to find out the role of sialidase in regulating this pathway in OC.

Anoikis is a type of apoptosis that occurs due to cell detachment from the extracellular matrix. Anoikis resistance is a critical feature in metastasis and aggressiveness of many cancers, including OC^22^. Anoikis-resistant cells retain both mesenchymal and epithelial markers with enhanced adhesion molecules like focal adhesion kinase (FAK) and integrin-β3 (ITG β3)^23–25^. Therefore, exploring the mechanism involved in anoikis resistance would be helpful to inhibit metastasis in the presence of sialidase.

Therefore, our overall aim was to explore the molecular interplay between autophagy, apoptosis, anoikis, and aberrant expression of the PI3K-Akt/mTOR pathway in the context of sialidase in OC. Initially, we observed a lower expression of cytosolic sialidase (Neu2) in OC cells with different mutations. Accordingly, we overexpressed Neu2 in these cells and demonstrated that it cleaved α2,6- and α2,3-linked SAs and reduced cell viability. Several autophagy-related molecules were also upregulated upon Neu2 overexpression. Atg5, a crucial protein for autophagosome formation, exhibited enhanced association with overexpressed Neu2 leading to its desialylation. Desialylated Atg5 now showed enhanced association both with Atg12 and Atg16L1 leading to more autophagosome formation and enhanced autophagy. We have also established that Neu2-induced apoptosis is through autophagy indicating their inter-relationship. Additionally, enhanced cytosolic Neu2 downregulated PI3K-Akt/mTOR pathway, which leads to upregulation of autophagy and anoikis. Our in vitro observations were further confirmed in the xenograft mouse model where intratumoral injection of Neu2 plasmid reduced the tumor mass. These tumor cells showed an enhanced association of Neu2 with Atg5 that leads to increased autophagosomes and apoptotic cells. Here we have provided evidence that Atg5 is a sialoglycoprotein and α2,6- and α2,3-linked SAs play a critical role in controlling autophagy/apoptosis both in vitro/in vivo in OC cells.

## Results

### Lower expression of Neu2 in OC cells

**Fig. 1:**
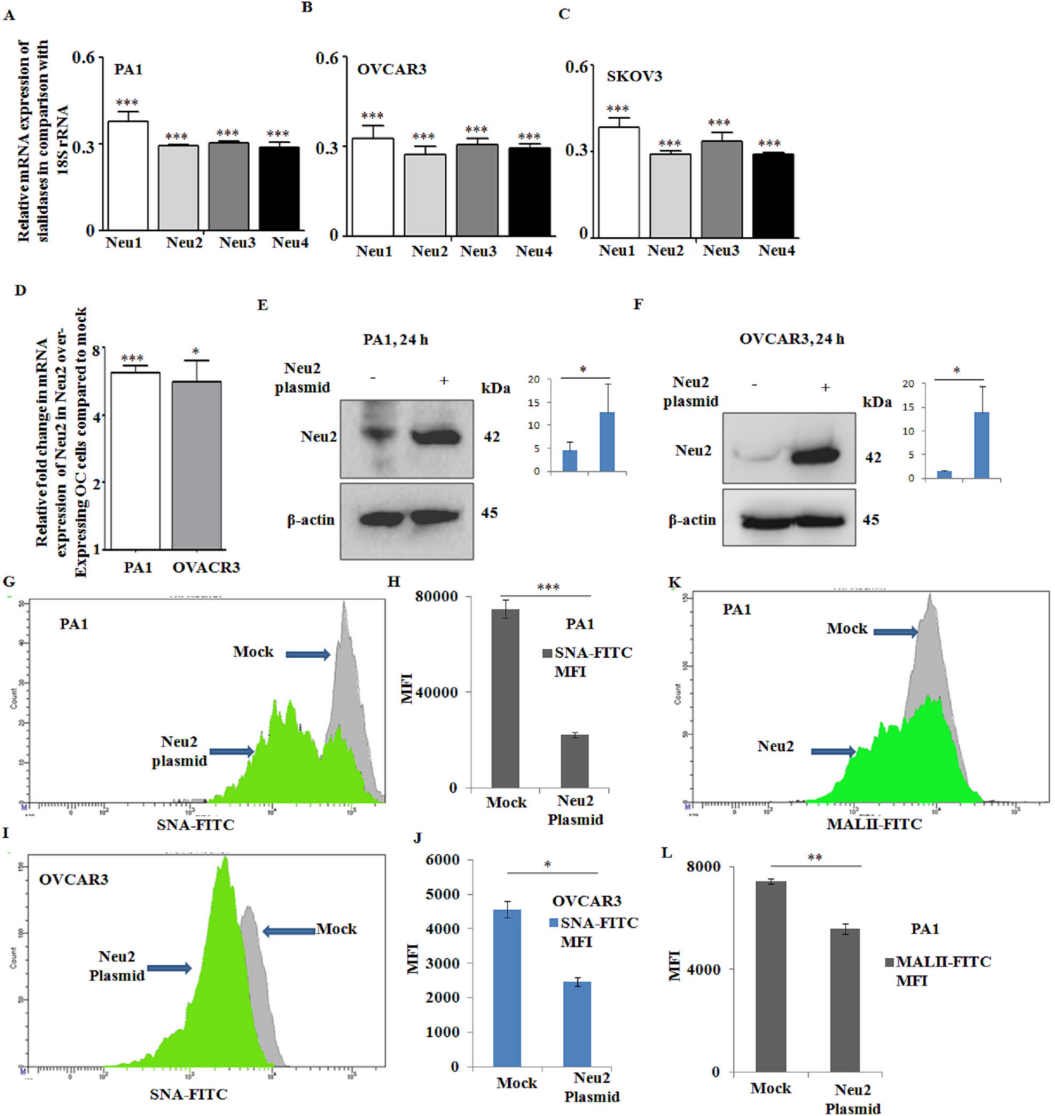
Overexpressed Neu2 demonstrated preferential specificity toward α2,6-linked sialic acids on OC cells. **A**–**C** Genetic expression levels of Neu1, Neu2, Neu3, and Neu4 in PA1 (**A**), OVCAR3 (**B**), and SKOV3 (**C**) ovarian cancer cell lines by real-time PCR analysis. Relative mRNA expression was expressed in comparison to 18S rRNA. **D** Relative fold change in the Neu2 mRNA level in PA1 and OVCAR3 after overexpression with Neu2 plasmid (24 h) compared to mock cells was analyzed by real-time PCR analysis. **E**, **F** Western blot analysis was performed to confirm the enhanced Neu2 protein levels in transfected PA1 (**E**) and OVCAR3 (**F**) cells compared to mock using an anti-Neu2 antibody. β-Actin was used as a loading control. **G**–**J** FACS analysis showing that Neu2 overexpression decreased the FITC-SNA binding in PA1 (**G**) and OVCAR3 (**I**) compared to mock cells. Bar graphs represent the MFI values of FITC-SNA-stained mock and Neu2-overexpressed PA1 (**H**) and OVCAR3 (**J**) cells. **K**, **L** Binding of FITC-MALII with mock and Neu2-overexpressed PA1 (**K**) by flow cytometry. The MFI values are compared by plotting a bar graph (**L**). The results are represented as mean ± SD from independent experiments and *p* values (*p < 0.05; **p < 0.01; ***p < 0.001; two-tailed *t* test) represented the significant differences between the means of the two test groups.

The role of mammalian sialidases (Neu1/Neu2/Neu3/Neu4) in OC is poorly understood. Initially, we evaluated the status of these sialidases in three different OC cells with dissimilar mutations by real-time PCR analysis where relative mRNA expression was measured in comparison to 18S rRNA. We have observed the significantly lower expression of cytosolic Neu2 in PA1 (Fig. 1A), OVCAR3 (Fig. 1B), and SKOV3 (Fig. 1C) in comparison with 18S rRNA, irrespective of the mutation status of these cells (Table S1). However, compared to Neu1, these differences between other sialidases are small. As Neu3 is specific for gangliosides, we were interested in Neu2 mainly due to its specificity toward both α2,6- and α2,3- linked SAs^26^. Accordingly, our main aim was to explore the context-specific role of Neu2 in OC. Therefore, we have transfected PA1 and OVCAR3 cells with Neu2 plasmid. The expression of Neu2 was confirmed by real-time PCR in comparison with mock cells (Fig. 1D). The overexpression of Neu2 in these cells after transfection was further validated by western blot analysis both in PA1 (Fig. 1E) and OVCAR3 (Fig. 1F).

### Comparing the presence of α2,6- and α2,3-linked SAs on OC cells

Initially, we checked the status of linkage-specific SAs on PA1 and OVCAR3 cells using fluorescein isothiocyanate (FITC)-conjugated *Sambucus nigra* agglutinin (SNA) and *Maackia amurensis* agglutinin (MALII), which binds to α2,6- and α2,3-linked SAs, respectively, by fluorescence-activated cell sorter (FACS). Binding of PA1 with SNA showed ~10-fold more binding compared to MALII suggesting the presence of more α2,6-linked SA on these PA1 cells (Table 1). Although drug-resistant OVCAR3 cells exhibited similar trends, however, it exhibited ~16-fold lower binding with both FITC-SNA and FITC-MALII compared to drug-sensitive PA1. Furthermore, we can conclude that the overall α2,3-linked SAs are less than α2,6-linked SAs in these OC cells (Table 1).

**Table 1:** Sialylation status of ovarian cancer cells.

Cell line	Linkage-specific lectin	Mock (MFI)	Neu2 plasmid (MFI)
PA1 (drug sensitive)	FITC-SNA	74,838	22,126
	FITC-MALII	7447	5591
OVCAR3 (drug resistant)	FITC-SNA	4565	2464
	FITC-MALII	455	425

Comparing the α2,6- and α2,3-linked sialic acids in mock and Neu2-plasmid-transfected PA1 and OVCAR3 cell lines using specific lectins as described in “Methods.”

### Enhanced Neu2 reduces cell surface sialylation

Neu2-overexpressed PA1 and OVCAR3 exhibited ~3.4- and ~1.9-fold decreased binding with SNA, respectively, as expressed in mean fluorescence index (MFI) compared to mock cells (Table 1 and Fig. 1G–J). These Neu2-overexpressing PA cells also showed decreased MALII binding compared to mock, MFI being 7447 compared to 5591. Therefore, the difference between Neu2 overexpression and mock in SNA binding with PA cells is much higher (MFI being 74,838 compared to 22,126) than MALII binding suggesting that, though Neu2 significantly cleaves both α2,3- and α2,6-linked SAs, it tends to show preferential specificity toward α2,6-linked SA than α2,3-linked SA present on the cell surface in these OC cells (Fig. 1K, L).

### Overexpression of Neu2 induces cell death and enhances autophagy

**Fig. 2:**
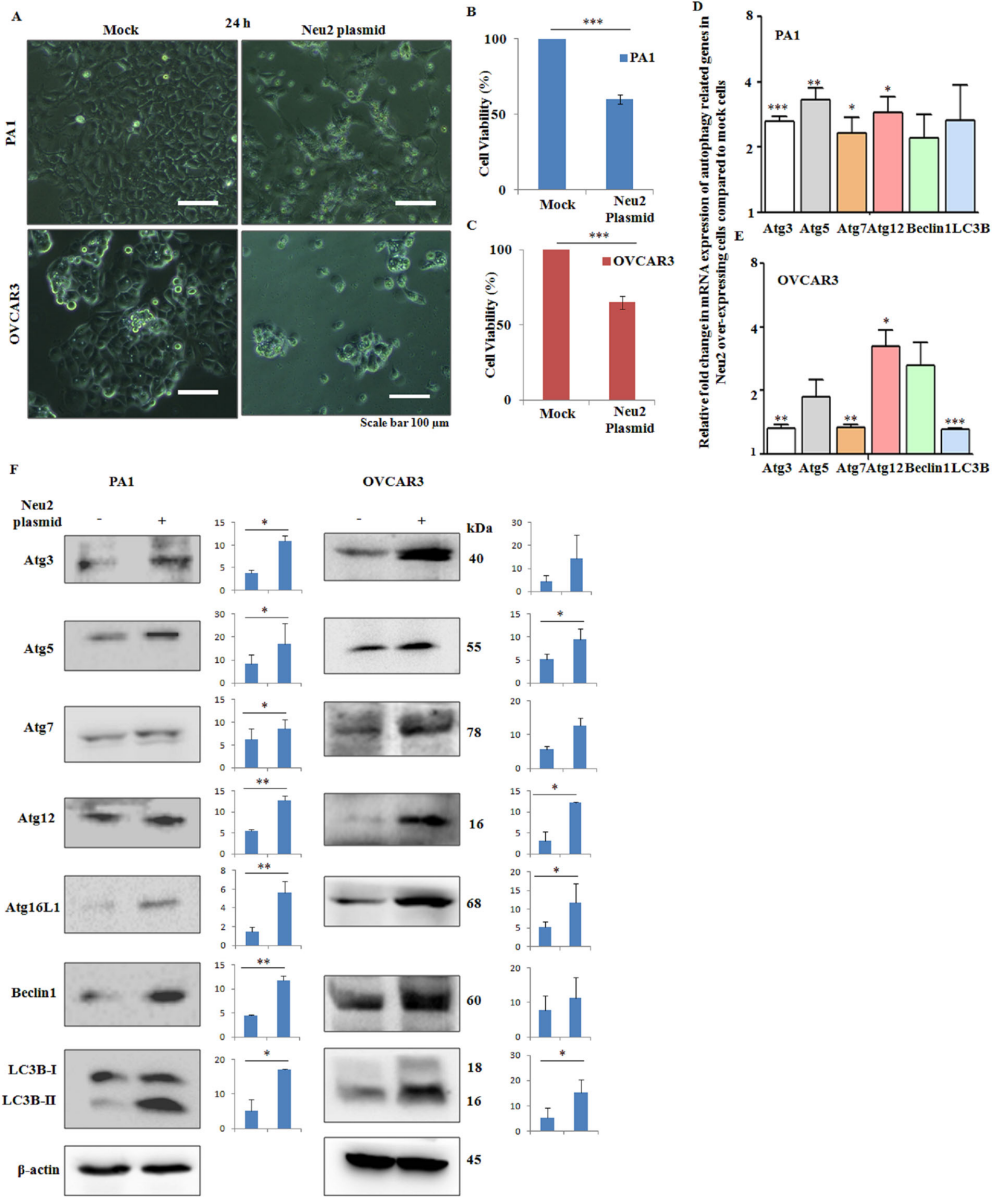
Neu2 transfection induced decreased cell viability and increased autophagy-related molecules. **A** Reduced size and changed shape of Neu2-transfected PA1 and OVCAR3 cells compared to mock were visualized through the phase-contrast image. **B**, **C** Decreased cell viability in both PA1 (**B**) and OVCAR3 (**C**) after Neu2 transfection as determined by MTT assay. **D**, **E** Representative bar graphs showing the mRNA expression levels of autophagy-related molecules in Neu2-overexpressed PA1 (**D**) and OVCAR3 (**E**) compared to mock by real-time PCR. **F** Western blots showing the protein levels of autophagy-related molecules after 24 h of Neu2 transfection compared to mock in these cells. β-Actin was used as a loading control. The results are represented as mean ± SD from independent experiments and *p* values (*p < 0.05; **p < 0.01; ***p < 0.001; two-tailed *t* test) represented the significant differences between the means of the two test groups.

Next, we found that Neu2 overexpression in both PA1 and OVCAR3 cells leads to changes in their morphology with reduced size and changed shape (Fig. 2A). Overexpressed Neu2 induced significant cell death up to ~40% in both PA1 (Fig. 2B) and OVCAR3 (Fig. 2C) within 24 h of transfection, as evidenced by 3-[4,5-dimethylthiazol-2-yl]-2,5 diphenyl tetrazolium bromide (MTT) assay.

Programmed cell death is classified into three types, type I or apoptosis, type II or autophagy, and type III or necrosis^27^. Therefore, we aimed to detect the type of Neu2-induced cell death in both PA1 and OVCAR3 cells. Initially, we checked the effect of Neu2 overexpression on autophagy. We observed higher mRNA expression levels of autophagy-related genes like Atg3, 5, 7, 12, Beclin1, and LC3B both in Neu2-overexpressing PA1 (Fig. 2D) and OVCAR3 (Fig. 2E) cells compared to mock cells. This was further confirmed in protein levels of Atg3, 5, 7, 12, 16L1, Beclin1, and LC3B-II by western blot analysis (Fig. 2F). All these observations suggest that enhanced Neu2 causes autophagy in OC cells.

### Enhanced association of overexpressed Neu2 with Atg5 leads to its linkage-specific desialylation causing increased autophagosome formation

**Fig. 3:**
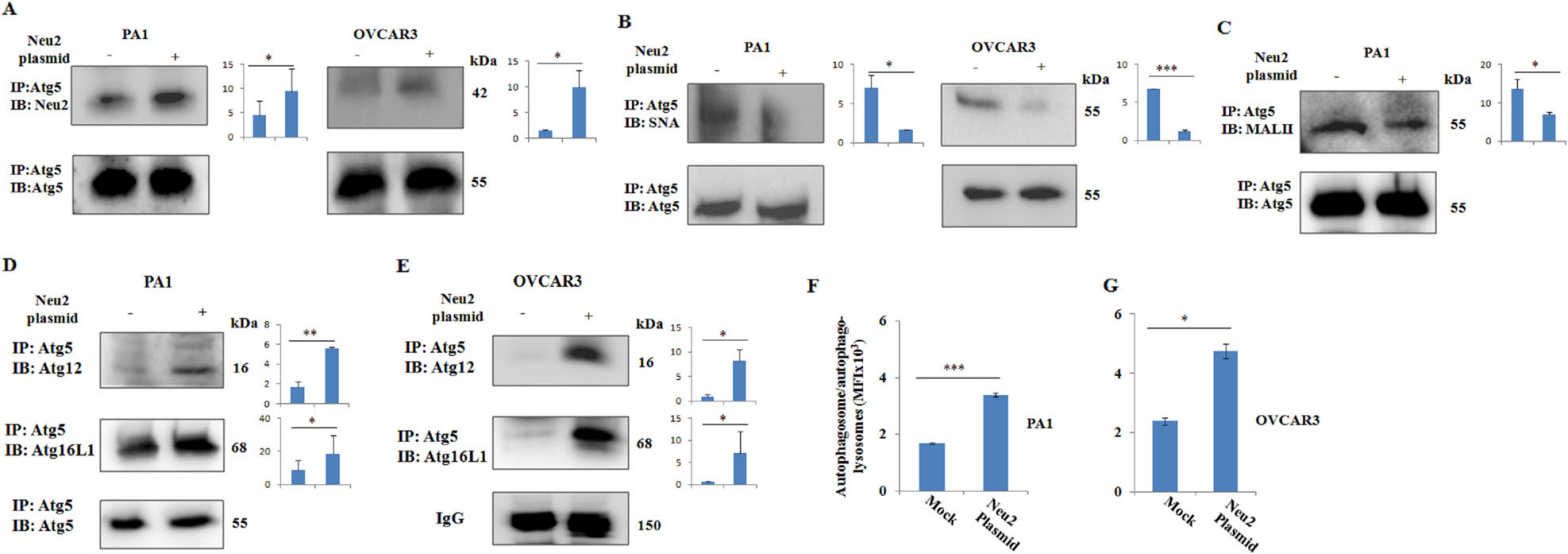
Linkage-specific desialylation of Atg5 by overexpressed Neu2 leads to enhanced autophagosome formation in OC cells. **A** Co-immunoprecipitation analysis showing the enhanced association of Atg5 with Neu2 in overexpressed conditions as compared to mock in both PA1 and OVCAR3 cells. Atg5 acts as a loading control. **B**, **C** Neu2 overexpression induced decreased binding of Atg5 with SNA in OC cells (**B**) and MALII in PA1 cells (**C**). Atg5 acts as a loading control. **D**, **E** Co-immunoprecipitation showing the increased association of Atg5 with Atg12 and Atg5 with Atg16L1 in Neu2-overexpressed PA1 and OVCAR3 compared to mock. Atg5 band and IgG band acts as the loading control in PA1 and OVCAR3, respectively. **F**, **G** Neu2-transfected PA1 (**F**) and OVCAR3 (**G**) cells showed enhanced autophagosomes/autophagolysosomes compared to mock as analyzed using specific green fluorescent dye by flow cytometry. The mean fluorescence index (MFI) is plotted on the bar graph for comparison. The results are represented as mean ± SD from independent experiments and *p* values (*p < 0.05; **p < 0.01; ***p < 0.001; two-tailed *t* test) represented the significant differences between the means of the two test groups.

So far, we have found enhanced Neu2-induced higher expression of autophagy-related molecules (Fig. 2). Autophagosome formation is a crucial step for autophagy, which essentially requires a complex Atg5-Atg12-Atg16L1 where Atg5 plays a major role that interacts covalently with Atg12 and non-covalently with Atg16L1^28^. Therefore, to understand the role of enhanced Neu2 in autophagosome formation, we have checked the association of Neu2 with Atg5 by co-immunoprecipitation (co-IP) assay. We have observed an enhanced association of Neu2 with Atg5 (Fig. 3A) in both Neu2-overexpressing PA1/OVCAR3 cells, which indicated the presence of SA on Atg5 protein. Next, we have observed the association of Atg5 with SNA, which confirmed that Atg5 is a sialoglycoprotein. Also, PA1 cells exhibited more α2,6-linked SA compared to OVCAR3 cells (Fig. 3B). However, Neu2-overexpressing PA1 and OVCAR3 cells exhibited reduced association of Atg5 with SNA confirming the desialylation of Atg5 compared to mock. PA1 cells also showed decreased α2,3-linked SA in the binding of Atg5 with MALII in Neu2-overexpressed condition (Fig. 3C). These experiments, for the first time, demonstrated that Atg5 is a sialoglycoprotein having both α-2,6 and α-2,3-linked SAs in OC cells.

More importantly, in the presence of enhanced Neu2, the desialylated Atg5 showed increased association with Atg12 as well as with Atg16L1 as analyzed by co-IP in both PA1 (Fig. 3D) and OVCAR3 (Fig. 3E). Therefore, overexpressed Neu2 induced linkage-specific desialylation of Atg5, which probably leads to its activation and subsequently preferred to form more Atg5-Atg12-Atg16L1 complex. Accordingly, we monitored the formation of autophagosomes using an autophagosome-specific fluorescence dye. A 2–3-fold enhanced autophagosome formation was observed in Neu2-transfected PA1 and OVCAR3 cells compared to mock (Fig. 3F, G). The next obvious question is whether this enhanced autophagy is responsible for inducing apoptosis in Neu2-overexpressed conditions in OC.

### Overexpressed Neu2 enhanced apoptosis in OC cells

**Fig. 4:**
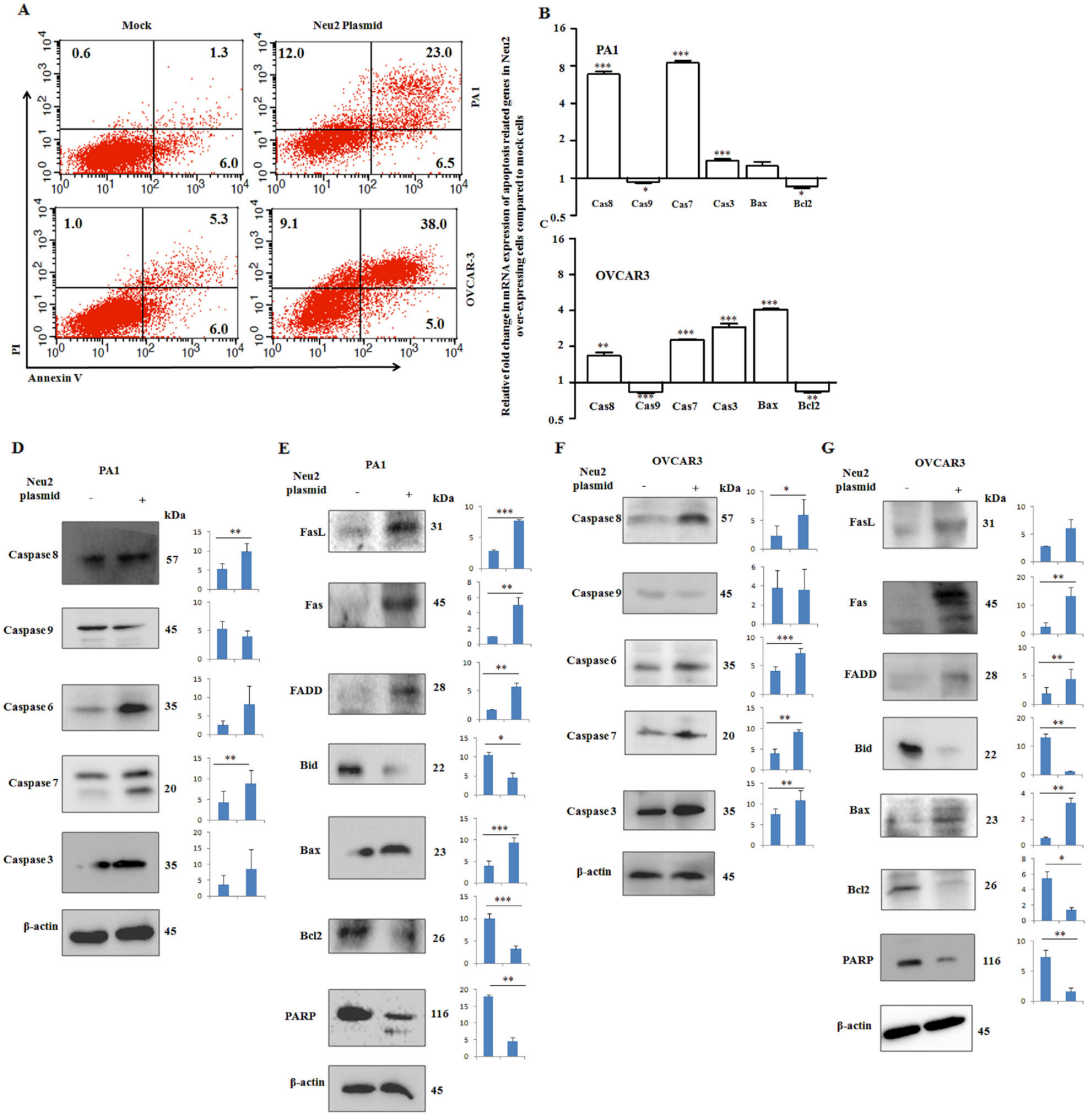
Neu2 transfection induced apoptosis in ovarian cancer cells. **A** Neu2-transfected PA1 and OVCAR3 cells were stained with Annexin-V/PI and analyzed by FACS and their apoptotic cell percent are presented and compared with mock. **B**, **C** mRNA levels of apoptosis-related molecules were analyzed by real-time PCR in Neu2-transfected PA1 and OVCAR3 cells compared to mock. **D**–**G** Western blot analysis showing the protein levels of apoptosis-related molecules in PA1 (**D**, **E**) and OVCAR3 (**F**, **G**) cells in mock and Neu2-transfected cells. β-Actin was used as a loading control. The results are represented as mean ± SD from independent experiments and *p* values (*p < 0.05; **p < 0.01; ***p < 0.001; two-tailed *t* test) represented the significant differences between the means of the two test groups.

We observed that Neu2 overexpression induces the externalization of phosphatidylserine in both OC cells, as evidenced by ~22 ± 5.5 and ~33 ± 7.0% increased Annexin-V positivity in PA1 and OVCAR3, respectively, compared to mock by FACS (Fig. 4A). This result indicates that overexpressed Neu2 induces ~3–4-fold enhanced programmed cell death in OC cells.

Next, we checked the several molecules involved in different apoptotic pathways in these cells. We observed upregulated mRNA expression of the initiator caspase (caspase 8) and effector caspases (caspase 3/7) in these Neu2-overexpressing cells compared to mock (Fig. 4B, C). Interestingly, there were no such changes observed in the mRNA level of caspase 9, an indicator of the intrinsic-mediated apoptotic pathway. Next, the total protein level of the caspase 6/7/8/9/3 was detected by western blotting (Fig. 4D, F). Both PA1 and OVCAR3 cells showed enhanced total protein levels of caspase 8, caspase 6, caspase 7, and caspase 3, whereas no such changes of caspase 9 were found in Neu2-overexpressed conditions. Accordingly, we checked different molecules involved in this pathway wherein Fas, FasL, and FADD were upregulated in Neu2-transfected OC cells (Fig. 4D–G). However, we have only been able to detect the significant reduction of uncleaved Bid protein, not the cleaved or truncated Bid, in Neu2-overexpressing OC cells. Poly ADP-ribose polymerase, a DNA repair enzyme, was also reduced along with an anti-apoptotic molecule Bcl2 in both Neu2-overexpressing OC cells compared to mock. The involvement of Neu2 in apoptosis was further validated by the upregulation of the pro-apoptotic molecule, Bax, both in genetic and protein levels (Fig. 4B, C, E, G). Therefore, our finding supports that Neu2 hinders cell proliferation by possibly affecting mainly through non-mitochondrial but caspase-associated extrinsic pathway without the involvement of caspase 9.

Induction of apoptosis by overexpressed Neu2 (Fig. 4) prompted us to check its consequence on the regulation of the cell cycle by comparing the expression of several kinases. We observed lower expression of several molecules involved in early G1 (cyclin-dependent kinase 4/6 (Cdk4/6)), late G1 (cyclin E/Cdk2), and G2-M (cyclin B1) phases in Neu2-transfected OC cells compared to mock (Fig. S1). Modulation of these cyclins and Cdks suggests that overexpressed Neu2-induced apoptotic signal halts the cell cycle in OC cells.

### Neu2 induces apoptosis through enhancing autophagy in OC cells

So far we have demonstrated Neu2-induced enhanced autophagy (Fig. 3) and apoptosis (Fig. 4) in OC cells. However, whether autophagy helps in the survival or death of cancer cells is a context-dependent mechanism^29^. Therefore, we aimed to explore that whether such enhanced autophagy is responsible for apoptosis in Neu2-transfected cells. To understand this, we treated both the OC cells with bafilomycin A1, a well-known autophagy inhibitor that prevents the autophagosome and lysosomal fusion step of autophagy.

**Fig. 5:**
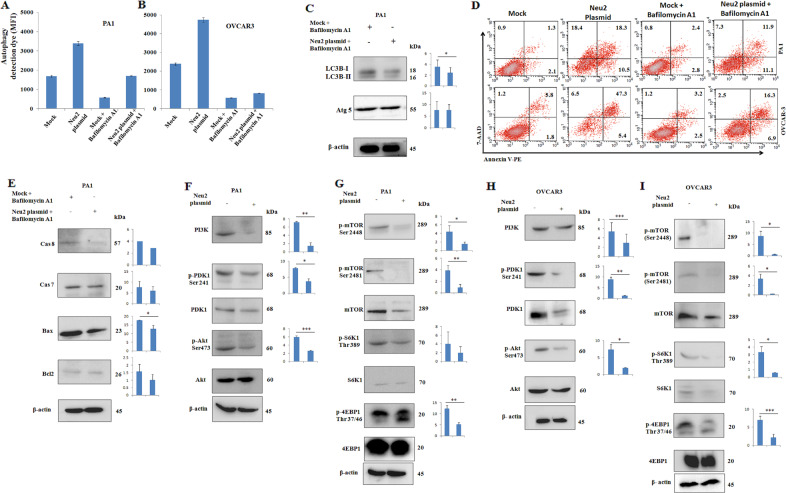
Neu2-enhanced autophagy leads to apoptosis via inhibiting PI3K-Akt/mTOR signaling pathway in OC cells. **A**, **B** PA1 and OVCAR3 cells were initially pretreated with bafilomycin A1 followed by Neu2 transfection in the presence of bafilomycin A1 for 24 h as described in “Methods.” Cells were stained with autophagy detection dye and analyzed by FACS; MFI presented as a bar graph for comparison. **C** LC3B and Atg5 levels were compared in treated cells (PA1) by western blot. β-Actin was used as a loading control. **D** These treated cells were stained with PE-Annexin-V/7AAD and analyzed by FACS. Percent of early and late apoptosis are indicated for comparison**. E** Western blots showed the differences in protein levels of apoptotic-related molecules (caspase8, 7, Bax, and Bcl2) in bafilomycin A1-treated mock and Neu2-overexpressing cells. **F**–**I** The protein levels of different PI3K-Akt/mTOR molecules were compared in mock and Neu2-transfected PA1 (**F**, **G**) and OVCAR3 (**H**, **I**) by western blot. β-Actin was used as a loading control. The results are represented as mean ± SD from independent experiments and *p* values (*p < 0.05; **p < 0.01; ***p < 0.001; two-tailed *t* test) represented the significant differences between the means of the two test groups.

As expected, bafilomycin A1-treated cells showed decreased autophagy both in PA1 (Fig. 5A) and OVCAR3 (Fig. 5B) as evidenced by FACS. Autophagy inhibition was further confirmed by decreased LC3B-II in Neu2-overexpressing PA1 in the presence of bafilomycin A1 (Fig. 5C). No change in Atg5 was found in these two conditions. Interestingly, bafilomycin A1 treatment significantly rescued the Neu2-overexpressing cells from apoptosis as evidenced by decreased Annexin-V/7-aminoactinomycin D (7AAD) positivity in PA1 (Fig. 5D). Similarly, bafilomycin A1-treated OVCAR3 also exhibited the reversal of such apoptosis. Reduced apoptosis was further corroborated by the decreased level of caspase 8, caspase 7, and Bax compared to mock in PA1 in the presence of bafilomycin A1 demonstrating the involvement of autophagy in Neu2-induced cell death (Fig. 5E). However, no change in Bcl2 level was observed under these conditions.

Beclin1, another important molecule, is mainly involved both in the initiation and fusion steps of autophagy^12^. Therefore, we knocked down Beclin1 in Neu2-transfected PA1. Again there was a rescue (~50%) from apoptosis as evidenced by reduced Annexin-V/propidium iodide (PI)-positive cells compared to Neu2 transfection alone (Fig. S2). All these observations conclusively demonstrated molecular crosstalk between enhanced autophagy and Neu2-induced apoptosis.

### Inhibition of PI3K-Akt/mTOR signaling pathway by Neu2 upregulation

To regulate cellular physiology, angiogenesis, and proliferation, PI3K-AKT/mTOR is one of the main pathways^30^. This pathway helps the cancer cell to grow and proliferate at a rapid rate and plays an important role both in autophagy and apoptosis in caffeine-treated cells^31^. So far, we have established a relationship between Neu2-induced autophagy and apoptosis (Fig. 5) in OC cells. Therefore, the next obvious question that remains to be addressed is whether this PI3K-AKT/mTOR pathway is also modulated in Neu2-overexpressed conditions. Here we observed lower expression of PI3K, phosphorylation of PDK1 at Ser241 as well as phosphorylation of Akt at Ser473, a downstream molecule of PDK1, after Neu2 overexpression in PA1 (Fig. 5F) and OVCAR3 (Fig. 5H) suggesting an involvement of this cytosolic sialidase in controlling this pathway.

Phosphorylation at Ser2481 and Ser2448 in mTOR leads to mTORC2 and mTORC1 complex formation, which has a significant contribution in the activation of the PI3K-AKT/mTOR pathway^32^. Upon Neu2 overexpression, both PA1 and OVCAR3 exhibited reduced phosphorylation leading to less mTORC1/C2 complex formation suggesting the involvement of Neu2 (Fig. 5G, I).

This reduced expression of mTORC1 leads to decreased phosphorylation of its downstream molecules S6K1 at Thr389 and 4E-BP1 at Thr37/46 in Neu2-overexpressing OC cells (Fig. 5G, I). All these observations demonstrated that overexpression of Neu2 may be responsible for the downregulation of several molecules in this pathway through their desialylation, which helps in enhancing autophagy thereby inducing apoptosis in OC cells.

### Enhanced autophagy sensitizes Neu2-overexpressing OC cells to detachment-induced cell death (anoikis)

**Fig. 6:**
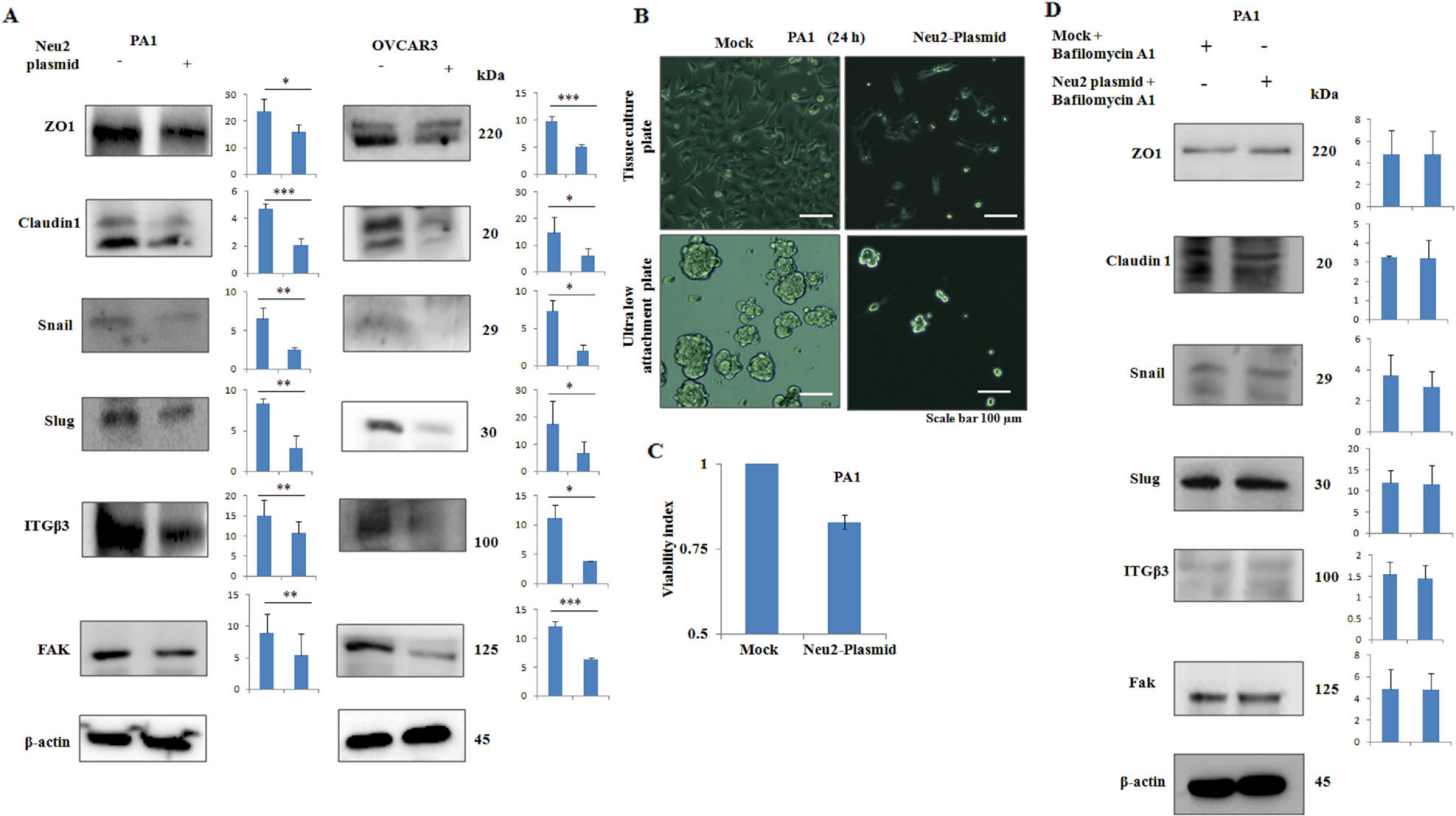
Neu2 induced enhanced autophagy promotes anoikis in OC cells. **A** Inhibition of epithelial, mesenchymal, and anoikis-resistant molecules in Neu2-transfected PA1 and OVCARC3 cells compared to mock by western blot analysis. β-Actin was used as a loading control. **B** Mock and Neu2-transfected PA1 cells were plated in adherent and non-adherent plates. Phase-contrast images were captured after 24 h to visualize the morphological changes. **C** The viability of these cells (as shown in **B**) was analyzed by MTT assay and the viability index was calculated as mentioned in “Methods” and represented in the bar graph. **D** Different EMT and anoikis-resistant molecules showed no significant difference between Neu2-transfected PA1 and mock after bafilomycin A1 treatment by western blot analysis. β-Actin was used as a loading control. Figures 5E and 6D western blots were performed under the same conditions using the same cell lysate, therefore the same beta-actin blot has been used as loading control in both the figures. The results are represented as mean ± SD from independent experiments and *p* values (*p < 0.05; **p < 0.01; ***p < 0.001; two-tailed *t* test) represented the significant differences between the means of the two test groups.

Anoikis resistance is an essential part of metastasis^22^. Next, we explored the involvement of overexpressed Neu2 in anoikis of OC cells. Neu2 transfection inhibited both epithelial (ZO-1 and Claudin-1) and mesenchymal (Snail and Slug) markers as well as anoikis-resistant molecules (ITG β3 and FAK) suggesting induction of anoikis both in PA1 and OVCAR3 (Fig. 6A). Additionally, we observed that Neu2 transfection leads to more cell death when growing in an ultra-low attachment (ULA) plate compared to a normal tissue culture (TCP) plate (Fig. 6B). The viability index (VI), which is the ratio of the percentage of live cells in ULA to TCP plates of mock and transfected cells, was calculated. VI <1.0 represents anoikis sensitization^23^. Here we observed the VI of 0.83 in Neu2-transfected cells compared to mock cells indicating induction of anoikis by Neu2 (Fig. 6C).

In general, autophagy assists anoikis resistance^12^. In contrast, glioma stem cells with silenced syndecan-binding protein reported higher levels of autophagy, which reduced anoikis resistance^33^. In this scenario, our next aim was to understand the role of enhanced autophagy in regulating anoikis in Neu2 overexpressed conditions in OC. Accordingly, we inhibited autophagy using bafilomycin A1. In this condition, overexpressed Neu2 exhibited minimal or no changes in the levels of epithelial, mesenchymal, and anoikis-resistant molecules suggesting that Neu2-enhanced autophagy sensitizes these cells to anoikis (Fig. 6D).

### Intratumoral injection of Neu2 plasmid reduced tumor size in murine xenograft model of human OC through increased autophagy

**Fig. 7:**
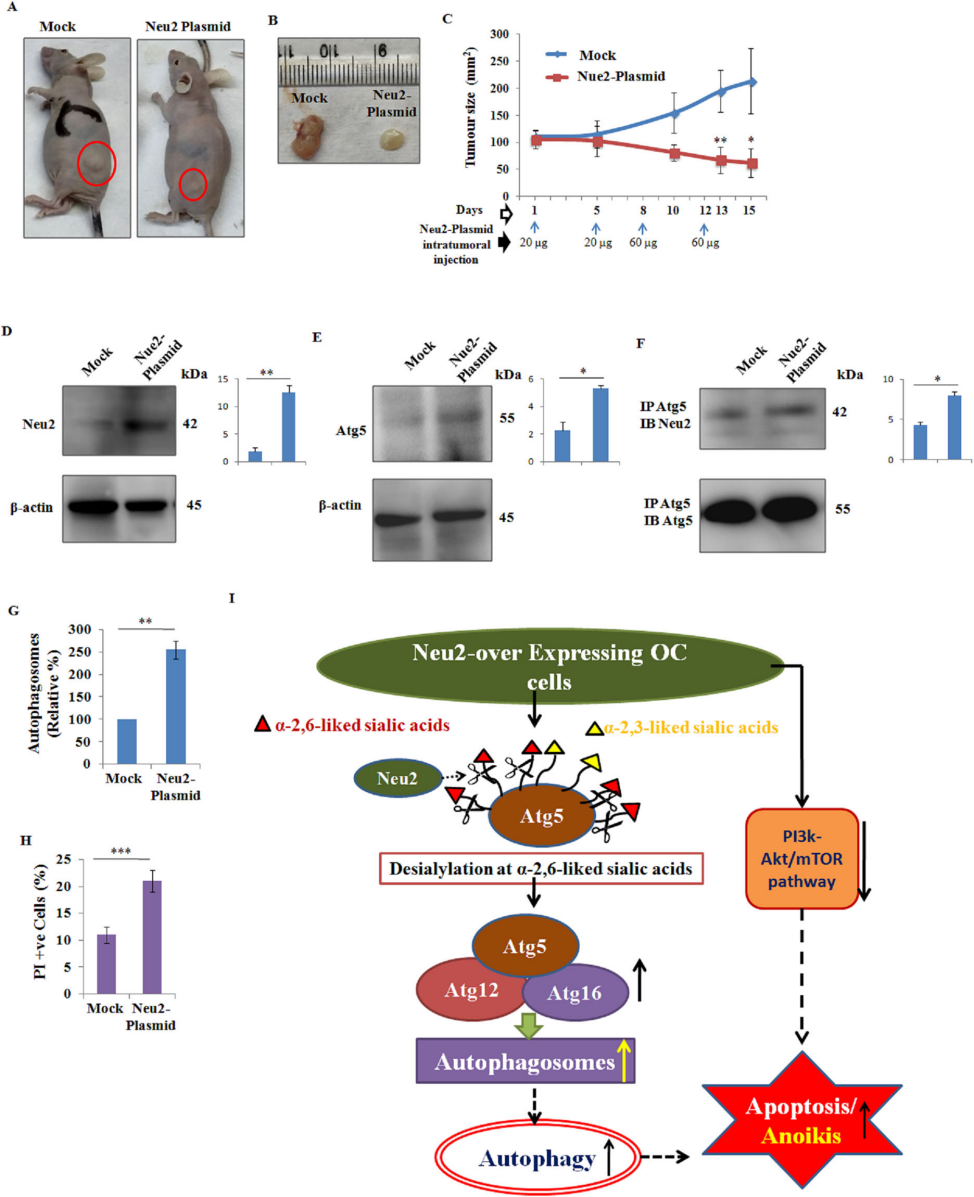
Neu2 plasmid decreased tumor size through enhancing autophagy and apoptosis in OC xenograft model. **A** PA1 cells (7 × 10^6^) in ~200 μl of PBS:matrigel (1:1) were injected into athymic Balb/c mice (*n* = 5) for generating human ovarian cancer xenograft. After tumor sizes reach ~80–120 mm^3^, Neu2 plasmid (1.0 μg/μl) was injected intratumorally for 4 times within 15 days as mentioned below. After 15 days of initial plasmid injection, images of all the experimental mice were captured and a representative image of a single mouse from each group is presented, highlighting the differences in tumor size between the two groups. **B** Tumors from mock and Neu2-plasmid-injected mice were isolated after 15 days of initial injection and representative images from both groups are shown**. C** In one group of human ovarian cancer xenograft mice (*n* = 5), 20 and 60 µg of Neu2 plasmid were injected intratumorally on days 1 and 5 and days 8 and 12, respectively. Concentration of plasmid injections given throughout the experiment and time points were mentioned on the *x*-axis and indicated with blue arrows. The tumor’s size (*y*-axis) was measured on days 1, 5, 10, 13, and 15 using a screw gauge and represented on the *x*-axis of the line graph. **D**, **E** Primary cells were isolated from these tumors and protein levels of Neu2 (D) and Atg5 (E) were analyzed by western blot; β-actin was used as the loading control. Representative blots are presented here. **F** Co-immunoprecipitation analysis of these proteins showed the enhanced association of Atg5 with Neu2 in Neu2-plasmid-injected tumors compared to mock. Representative blots are presented here. **G** Primary cells from these tumors were stained with autophagy-specific dye and analyzed by FACS. Mock cells MFI value was considered as 100% and Neu2-plasmid cells MFI was compared on bar graph. **H** These cells were also probed with PI and processed similarly. The results are represented as mean ± SD from independent experiments and *p* values (*p < 0.05; **p < 0.01; ***p < 0.001; two-tailed *t* test) represented the significant differences between the means of the two test groups. **I** Schematic diagram outlining the overexpressed Neu2 and sialidase-induced desialylation of Atg5 at α2,6 linkage leading to its activation to form increased Atg5-Atg12-Atg16L1 complex, which produced more autophagosomes. Neu2 induced enhanced autophagy ultimately promotes anoikis/apoptosis. Neu2 overexpression also inhibits the PI3K-Akt/mTOR pathway, responsible for such apoptosis in OC cells.

To further confirm our in vitro observations, we generated a xenograft model of OC by subcutaneously injecting PA1 into athymic mice (Fig. 7A). Neu2 plasmid was injected intratumorally four times. Tumors were isolated after 15 days of initial plasmid injection and tumor size was compared. Neu2-plasmid-injected mice showed reduced tumor size (~4–5-fold) compared to mock (Fig. 7B, C).

The primary cells isolated from these tumors showed increased Neu2 and Atg5 levels in Neu2-plasmid-injected tumors (Fig. 7D, E). We also observed more association of increased Atg5 with this enhanced Neu2 (Fig. 7F) leading to its desialylation. As expected, enhanced autophagy was also observed as evidenced by increased autophagosomes in Neu2-plasmid-injected tumors. This again confirms the contribution of desialylated Atg5, the most important autophagy molecule in enhancing autophagosomes (Fig. 7G). Finally, these cells showed enhanced PI positivity indicating that Neu2 induced enhanced cell death by FACS (Fig. 7H). All these results demonstrated the physiological relevance of our study, which corroborated with our in vitro experiments.

## Discussion

The main achievement of this study is to demonstrate the role of overexpressed sialidase (Neu2) in regulating autophagy in OC cells. Additionally, we demonstrated the role of overexpressed Neu2 both in apoptosis and anoikis through the induction of autophagy in these cancer cells. More importantly, this is the first report showing that Atg5 is a sialoglycoprotein having both ɑ2,3- and ɑ2,6-linked SAs. This sialoglycoprotein showed a close association with overexpressed Neu2, and thereby it causes desialylation by removing its ɑ2,6- and ɑ2,3-linked SAs. Desialylated Atg5 is now activated and showed more interaction both with Atg12 and 16L1 that leads to increased autophagosome formation. Furthermore, overexpressed Neu2 induced more autophagy-related molecules and caused increased autophagy. Additionally, we demonstrated that enhanced autophagy sensitizes OC cells to anoikis in Neu2-overexpressed condition. We also observed that overexpressed Neu2 inhibited many molecules in the PI3K-Akt/mTOR pathway, which are aberrantly expressed during cancer progression. Under this experimental condition, this signaling event is possibly modulated through desialylation of all these or a few selected signaling molecules. Therefore, desialylation possibly is a necessary step, which probably plays a very important role in helping cancer regression.

Increased sialylation is one of the hallmarks to show the aggressiveness of many cancers, including OC^2,4,34^. Furthermore, elevated levels of SAs in serum are also correlated with the clinically used diagnostic marker CA-125 in advanced-stage OC^5^. Here we observed that even the OC cells exhibited enhanced SAs. Interestingly, α-2,6-linked SAs were predominantly present on these cancer cells compared to α-2,3-linked SAs. Therefore, we are very much hopeful that estimation of linkage-specific SAs probably would be a better biomarker than total SAs in patient serum for diagnosis or monitoring in near future.

The presence of a higher amount of SAs was corroborated by the significantly lower expression of Neu2 in different types of OC cells with various mutations. In general, Neu2 showed its specificity toward both α2,6- and α-2,3-linked SAs^26^. Therefore, it is very important to understand the role of Neu2 in OC cells, which could be helpful for target identification.

The basal level of autophagy is necessary for cell survival^29^. It is an essential process required for catabolism of sialyloligosaccharides in normal mouse embryonic fibroblasts cells^35^. However, a higher level of autophagy is toxic^36^. Therefore, enhancing autophagy might be a helpful strategy to induce cell death in OC. As we observed higher sialylation and lower Neu2 expression in OC cells, we explored the connection between sialylation and autophagy through the involvement of cytosolic sialidase (Neu2). We found Neu2 overexpression enhanced different autophagy-related molecules in these cancer cells.

Atg5 is an essential molecule for initiating the autophagosome formation process^28^. Defects in autophagosome formation, as well as accumulation of sialyloligosaccharides, were reported in Atg5 knocked out normal fibroblasts cells^35^. Increased Atg5 in Neu2-overexpressed condition hinted us a possible interaction of this enzyme with Atg5. To be sure, we found the enhanced association of Atg5 with Neu2 leading to its desialylation. We also demonstrated that the desialylation of Atg5 is an essential step for its activation, which is crucial in forming the Atg5-Atg12-Atg16L1 complex and also a decisive step in autophagosome formation confirming the role of overexpressed Neu2 in the enhancement of autophagy.

Earlier, we demonstrated that Neu2 induces apoptosis through the extrinsic pathway in pancreatic cancer cells^19^ and also in pancreatic cancer stem-like cells^11^. Here also, we observed a similar mode of apoptosis in Neu2-overexpressed OC cells. Therefore, it may be envisaged that Neu2, in general, preferred to induce apoptosis through extrinsic pathway in cancer cells possibly independent of the type of cancer.

Autophagy and apoptosis are interconnected: enhanced autophagy may suppress or enhance the apoptosis^37^. Autophagy-induced apoptosis by digitoxin was reported in lung cancer cells^38^. Here we found that Neu2-induced enhanced autophagy leads to apoptotic cell death in OC preferentially through the extrinsic pathway. Here we have detected only the total caspase 9 level at 47 kDa. However, no cleaved bands were detected for caspase 9 in Neu2-overexpressing OC cells. This observation is corroborated by other research articles wherein leptospire-infected macrophages^39^, Hh003-treated colon, and pancreatic cancer cells^40^ undergo caspase 9-independent extrinsic pathway-mediated apoptosis. A similar trend was also observed in the Bid expression level, where a significant reduction of uncleaved Bid was observed in OC cells after Neu2 overexpression. However, we were unable to detect cleaved or truncated Bid in Neu2-overexpressing OC cells. Our observation is in agreement with a couple of reports, where the TRAIL-mediated apoptosis in human osteosarcoma cells^41^, nasopharyngeal carcinoma^42^, and mahanine-mediated apoptosis in human leukemic cells^43^ showed the reduction in the level of expression of Bid protein, which has been mentioned as the cleavage of Bid. Thus a reduction in Bid expression level in Neu2-overexpressing cells suggests Neu2-induced Bid cleavage. Therefore, it may be envisaged that Neu2-overexpressing cells undergo apoptosis through the extrinsic pathway without caspase 9 involvement in non-mitochondrial mode. Moreover, in the presence of autophagy inhibitors, most of the Neu2-overexpressing cells were rescued from apoptosis demonstrating a direct role of autophagy in inducing apoptosis.

Caffeine-induced apoptosis by enhancement of autophagy through inhibition of PI3K/Akt/mTOR pathway is reported in HeLa cells^31^. Therefore, this pathway plays a crucial role in both apoptosis and autophagy. Higher sialylation activates the PI3K-AKT/mTOR pathway in cancer^19,21^. We believe that the desialylation of several proteins involved in this pathway is responsible for its inhibition leading to enhanced autophagy as well as apoptosis in Neu2-overexpressed OC cells demonstrating the important role of this sialidase.

Anoikis resistance, responsible for metastasis, is well known in highly aggressive OC cells^22^. Simultaneous increase of both epithelial and mesenchymal markers is an indication of anoikis resistance^23^. Interestingly, we observed decreased epithelial, mesenchymal, and adherent molecules in the Neu2-overexpressed condition suggesting induction of anoikis in these OC cells. This was corroborated by more cell death even in ULA plates. However, desialylation of all these or a few selected molecules involved in Neu2-induced anoikis sensitization demands further in-depth investigation.

Autophagy is an energy recycling process. It generally assists cancer cells in anoikis resistance^12^. The contrasting report is also available wherein glioma stem cells exhibited enhanced autophagy, which reduced anoikis resistance only when syndecan-binding protein was silenced^33^. Here we have convincingly demonstrated overexpressed Neu2 as an autophagy inducer, which in turn sensitizes OC cells to anoikis indicating a close relationship between these two processes.

All these in vitro observations were corroborated by our in vivo studies. Tumor cells isolated from Neu2-plasmid-injected human OC xenograft mice also showed the increased association of Neu2 with Atg5 leading to its desialylation, enhanced autophagosomes, and increased cell death, ultimately leading to a reduction in tumor size. Taken together, our experimental evidence establishes a crucial role of Neu2 in demonstrating a connection between enhanced autophagy and anoikis/apoptosis in OC.

## Conclusion

Taken together, we can summarize that Neu2 may be considered as an alternative therapeutic strategy for the management of OC. Neu2 overexpression causes desialylation of ɑ2,6- and ɑ2,3-linked SAs that are present on Atg5 protein in these cancer cells. Desialylated Atg5 helps in forming more autophagosomes. This enhanced autophagy by Neu2 is closely associated with anoikis/apoptosis in OC. All these events act together and possibly are responsible for tumor regression. A pictorial representation of this study has been demonstrated in Fig. 7I.

## Methods

### Reagents and antibodies

All the primary antibodies, ITG β3 (13166), FAK (3285), rabbit immunoglobulin G (IgG), horseradish peroxidase (HRP)-linked antibody (7074), β-actin (4970), autophagy antibody kit (4445), EMT Antibody Kit (9782), and SignalStain® DAB Substrate Kit (8059) were from Cell Signaling Technology. FITC-Annexin-V (556547), BD Cycletest™ Plus DNA Kit (#340242), and ULA 96-well plates were from BD Bioscience. ChemiDoc MP imaging system was from BIO-RAD and Azure c400 Visible Fluorescent Western Blot Imaging System was from Azure Biosystems. Cell culture medium RPMI-1640, Minimum Essential Medium (MEM), fetal bovine serum (FBS), antibiotic–antimycotic, trypsin–EDTA, Anti-Neu2 antibody (MA525555), H_2_DCFDA (C6827), Lipofectamine 2000, LTX, and Plus reagent were from Invitrogen, USA. MTT, anti-LC3B antibody (L7543), PI, molecular grade bovine serum albumin, Tween-20, dimethyl sulfoxide (DMSO), collagenase, matrigel, and bafilomycin A1 and other chemicals were from Sigma-Aldrich, USA. Autophagy detection kit (139484) was from Abcam. BCA protein assay kit, West pico-ECL system and Beclin1 siRNA (Assay ID:137199, Catalog#AM16708), RevertAid First Strand cDNA Synthesis Kit (K1622), and Maxima SYBR Green qPCR Master Mix (2X; K0251) were from Thermo Fisher Scientific, USA. Polyvinylidene difluoride (PVDF) membrane was from MILLIPORE, Bedford, MA, USA (Immobilon-P PVDF Membrane# IPVH00010). SNA (B-1305) and MALII (B-1265) were from Vector Laboratories, USA.

### Cell cultures

Human OC cell lines PA1, OVCAR3, and SKOV3 were purchased from the National Centre for Cell Science cell repository, Pune. PA1 was grown in MEM and OVCAR3/SKOV3 in RPMI-1640 supplemented with 10% FBS, glutamine (2.2 g/l), and 1% antibiotic–antimycotic (complete medium) at 37 °C with 5% CO_2_. Mutation status of these three cell lines is mentioned in Supplementary Table 1. The cell lines were authenticated by STR profiling, and the mycoplasma test was negative.

### Real-time PCR analysis

RNeasy Mini Kit was used for extracting RNA from cells^44^. First-Strand cDNA was synthesized with the Revert Aid Kit using specific primers (Table S2). Real-time PCR was performed using a Maxima SYBR Green qPCR Master Mix. LightCycler 96 software was used for the quantification of relative amounts of target mRNA. 18S rRNA was used as the internal control.

### Transfection

Lipofectamine LTX, plus reagents were used for transfection^45^. In brief, cells (5 × 10^5^) were plated in a 6-well plate with Opti-MEM (800 μl) for 24 h. Plus reagent and Neu2 plasmid DNA (1.5 μg) or Beclin1 siRNA (3 μg) were taken in a 1:1 ratio with Opti-MEM (100 μl) and kept for 15 min at 25 °C. LTX (4 μl) in Opti-MEM (100 μl) was added and incubated for 30 min. This mixture (200 μl) was added to each well and incubated for 6 h. Cells were cultured with the fresh complete medium for another 24 h. RNA and protein were isolated for real-time PCR and western blot analysis, respectively.

### Identification of linkage-specific SAs by FACS

The status of linkage-specific sialylation was determined using two SA-binding lectins, namely, SNA and MALII^46^. Mock and Neu2-transfected cells (1 × 10^5^) cells were washed and resuspended in lectin-binding buffer (20 mM Tris, 0.5 M NaCl, 2.0 mM MnCl_2_, 2.0 mM MgCl_2_, 2.0 mM CaCl_2_). FITC-conjugated SNA and MALII (5.0 μg/ml at 4 °C) were incubated separately with cells for 1 h. The positivity of FITC was acquired by FACS with the CellQuestPro software.

### Western blot

OC cells (1 × 10^6^) were sonicated (Qsonica-LLC, XL-2000 series, Newtown, CT, USA) and then cell debris was removed by centrifugation (10,000 × *g*, 10 min at 4 °C)^47^. The supernatants were collected and the proteins were quantified with the BCA protein assay kit. Sodium dodecyl sulfate-polyacrylamide gel electrophoresis (SDS-PAGE; 5–12%) was used for the separation of proteins according to their molecular weights and then it was electrotransferred to the PVDF membrane. Protein bands were detected by the West-pico ECL system and images were captured by Biorad ChemiDoc MP System (Bio-Rad, Hercules, CA, USA) and also by Azure c400 Western Blot Imaging System. β-Actin was used as a loading control. Primary cells from mouse tumors were also processed similarly for specific proteins.

### Co-immunoprecipitation

Cell lysate (200 μg/150 μl) was incubated with an anti-Atg5 antibody (1:100) overnight at 4 °C^48^. The immuno-complex was incubated with protein A-sepharose 4B for 3 h at 4 °C and washed with ice-cold phosphate-buffered saline (PBS). The immune complex was resuspended in SDS-PAGE sample buffer without β-mercaptoethanol (reducing agent) and then separated by SDS-PAGE (10%). Molecules were identified using specific antibodies to check the association of Atg5 with Neu2, Atg12, and Atg16L1. Secondary antibody rabbit IgG was used for all the blots to develop the bands. In the case of co-IP, the developed non-specific IgG band indicated the equal pull-down thus used as a loading control.

Immuno-complex was also incubated with biotinylated SNA and MALII and then developed with avidin–HRP to detect the status of α2,6- and α2,3-linked SAs on Atg5. The cells isolated from mouse tumors were similarly processed.

### Cell viability assay

Neu2-induced cell death was determined by the MTT assay^49^. Briefly, mock and Nue2-transfected PA1/OVCAR-3 (4–8 × 10^3^ ×/250 µl/well) cells were incubated at 37 °C for 24 h in 96-well plates. These cells were visualized under a phase-contrast microscope. Additionally, the plate was centrifuged at 1000 rpm for 10 min at 4 °C and the medium was discarded. MTT, dissolved in Iscove’s Modified Dulbecco’s Medium (IMDM), 100 µg/well, was added and incubated for 3 h at 37 °C. Formazan crystals were dissolved in DMSO and quantified at 550 nm in an enzyme-linked immunosorbent assay plate reader and percentage of cell viability was calculated.

### Quantification of autophagosomes

Briefly, Neu2-transfected and mock PA1 (1 × 10^6^) cells were harvested, washed, and resuspended in PBS–5% FBS^13^. Then green detection reagent from the autophagy detection kit was added and incubated at 37 °C for 30 min. Cells were washed and analyzed by FACS. OVCAR3, primary cells from mouse tumors from each group, and bafilomycin A1-treated cells were processed similarly.

### Detection of externalized surface phosphatidylserine by flow cytometry

Neu2-induced apoptosis was determined by suspending mock, PA1-transfected, or OVCAR3-transfected (5 × 10^5^) cells in Annexin-V-binding buffer and incubated for 30–45 min at room temperature as described earlier^50^. FITC-Annexin-V/PI (1 µg/ml) or phycoerythrin-Annexin-V/7AAD [5 µl (0.25 µg)/test] were added and incubated for 20 min in the dark at 4 °C. These samples were analyzed by FACS.

### Bafilomycin A1 treatment in OC cells

Adhered PA1 and OVCAR3 cells (5 × 10^5^) in the 6-well plate were preincubated separately with bafilomycin A1 (100 nM) for 2 h. Bafilomycin A1 was replaced with transfection medium (mock or Neu2) and incubated for 6 h. The transfection medium was replaced with fresh medium containing 20 nM bafilomycin A1 and incubated up to 24 h and analyzed both by FACS and western blot as described above.

### Anoikis assay

Equal numbers of mock and Neu2-transfected PA1 (1 × 10^4^) were seeded both in normal (TCP) and ULA 96-well tissue culture plates and incubated at 37 °C for 24 h and MTT assay was performed^13^ and VI was calculated. Additionally, phase-contrast images were taken to compare TCP and ULA plates.

### Xenograft murine model

Female athymic BALB/c mice (*n* = 5, 4–6 weeks old) were used for the xenograft murine model^51^. PA1 (7 × 10^6^) in ~200 μl of PBS:matrigel (1:1) were injected subcutaneously. Tumors (~80–120 mm^3^) were generated within 20 days and monitored using a screw gauge.

Neu2 plasmid (2.5 μg/μl, 80 μl in water), lipofectamine 2000 (120 μl), and Opti-MEM medium (40 μl) were mixed and incubated for 30 min at 25 °C to yield a transfection mixture with plasmid concentration (1.0 μg/μl). This (20 μl) was injected intratumorally using a syringe with 31 G needles on days 1 and 5. Subsequently, the Neu2 transfection mixture (60 μg) was again injected on days 8 and 12. Transfection mixture without plasmid was used similarly, which served as control (mock). These mice were sacrificed on day 15 and the tumor was isolated.

Tumor tissues were dissected in small pieces (~3 mm^3^) with a scalpel and treated with collagenase solution (1 mg/ml in IMDM)^43^. It was incubated for 2 h at 37 °C on a shaker (30 rotations/min), centrifuged, washed, and resuspended in the medium. Cells were used on the same day for subsequent experiments.

The sample size was five for each group, among which none was excluded. No randomization was used. The researchers were not blinded to the experiments. All procedures were approved by the institutional animal ethical committee (CSIR-IICB-AEC) on animal experimentation (Ref No. IICB/AEC/Meeting/2016/October; dated: 31.10.2016).

### Statistical analysis

The data shown are representative of three sets of independent experiments. Each experiment was performed in triplicate and all the western blots were quantified using the ImageJ software. The results were analyzed using GraphPad Prism (version 5.1). Two-tailed Student’s *t* test was used to detect the statistical differences between the groups. Standard error bars represent the standard deviation of the mean (±SD) and *p* values (*p < 0.05; **p < 0.01; ***p < 0.001) represented the significant differences between the means of the two test groups. The sample size was chosen as per the well-established rules in the literature and according to our previous experience.

## Supplementary information

Supplementary Figure legends

Table S1 Information on human ovarian cancer cell lines

Table S2 Primers used to check genetic expression of different molecules by real time PCR

Figure S1 Overexpressed Neu2 halts the cell cycle in ovarian cancer cells.

Figure S2 Beclin-1 knockdown reduced the Neu2-induced apoptosis in ovarian cancer cells.
