# Supplementary material for: Desialylation of Atg5 by sialidase (Neu2) enhances autophagosome formation to induce anchorage-dependent cell death in ovarian cancer cells
Source: Cell Death Discov. 2021 Feb 1;7:26. doi: 10.1038/s41420-020-00391-y (PMC7851153; doi:10.1038/s41420-020-00391-y)
Supplement: Supplementary file 1 — Supplementary Figure legends [file 41420_2020_391_MOESM1_ESM.docx]

**Supplementary Figure legends**

***Figure S1.*** *Overexpressed Neu2 halts the cell cycle in ovarian cancer cells.*

Protein levels of cell cycle-related molecules were compared between mock and Neu2-transfected PA1 and OVCAR3 cells by western blot. β-actin used as control.

***Figure S2*** *Beclin-1 knockdown reduced the Neu2-induced apoptosis in ovarian cancer cells*

Cells were co-transfected with both Neu2 and Beclin1 siRNA or only with Neu2 and analyzed for annexin V/PI positivity after 24 h by FACS**.**

***Table S1*** *Information on human ovarian cancer cell lines*

Part of the information has been given in our earlier report**^13^.**

***Table S2*** *Primers used to check genetic expression of different molecules by real time PCR*

All these information has also been mentioned in our earlier papers**^13, 19^**.
