# Supplementary material for: Desialylation of Atg5 by sialidase (Neu2) enhances autophagosome formation to induce anchorage-dependent cell death in ovarian cancer cells
Source: Cell Death Discov. 2021 Feb 1;7:26. doi: 10.1038/s41420-020-00391-y (PMC7851153; doi:10.1038/s41420-020-00391-y)
Supplement: Supplementary file 2 — Table S1 Information on human ovarian cancer cell lines [file 41420_2020_391_MOESM2_ESM.docx]

***Table S1*** *Information on human ovarian cancer cell lines*

| **Cells derived from** **ascitic fluids** | **Disease type** | **Genetic status** | |
| --- | --- | --- | --- |
|  |  | **P53** | **RAS** |
| PA1 | teratocarcinoma | WT | N-Ras mutated |
| OVCAR-3 | adenocarcinoma | mutated | WT |
| SKOV3 | adenocarcinoma | mutated | WT |

Part of the information has been given in our earlier report **[13].**
