# Supplementary material for: Desialylation of Atg5 by sialidase (Neu2) enhances autophagosome formation to induce anchorage-dependent cell death in ovarian cancer cells
Source: Cell Death Discov. 2021 Feb 1;7:26. doi: 10.1038/s41420-020-00391-y (PMC7851153; doi:10.1038/s41420-020-00391-y)
Supplement: Supplementary file 3 — Table S2 Primers used to check genetic expression of different molecules by real time PCR [file 41420_2020_391_MOESM3_ESM.docx]

***Table S2*** *Primers used to check genetic expression of different molecules by real time PCR*

| ***NAME*** | ***FORWARD PRIMER*** | ***REVERSE PRIMER*** |
| --- | --- | --- |
| Neu1 | GGAGCAAGGATGATGGTGTT | CATGATCATCGCTGAGGAGA |
| Neu2 | CCTCTTCTTCATTGCCATCC | GTCGTGAAGCTGCAAACAAT |
| Neu3 | CAGATTGTGTCAGGCAGGAA | AGCTGGAAGCAAAAGAACCA |
| Neu4 | CACCGTCTTCCTCTTCTTCA | GGCAGATCTTGCCAAAACA |
| Caspase 9 | TAGAAAACCTTACCCCAGTG | TCAGGATGTAAGCCAAATCT |
| Caspase 8 | AGACTGATTCAGAGGAGCAA | GTGACTGGATGTACCAGGTT |
| Caspase 7 | GATTTGACAGCCCACTTTAG | TTCCACTGGGATCTTGTATC |
| Caspase 3 | AGGAGCAGTTTTGTTTGTGT | CAGGCCTGAATAATGAAAAG |
| Bax | TCACTGAAGCGACTGATGT | GAGGAAAAACACAGTCCAAG |
| Bcl2 | ATTGTGGCCTTCTTTGAGT | TACAGTTCCACAAAGGCATC |
| GAPDH | GTCAGGTCCACCACTGACAC | GGAAGGACTCATGACCACAG |
| LC3B | GGTGAGAAGCAGCTTCCTGT | TCTCCTGGGAGGCATAGACC |
| Beclin1 | GGACACTCAGCTCAACGTCA | AGCCTGGACCTTCTCGAGAT |
| Atg3 | GAGCAACGGCAGCCTTTAAC | TCCAAGTTCTCCCCCTCCTT |
| Atg5 | ACAGATGACAAAGATGTGCT | TGGTGTGCCTTCATATTCAA |
| Atg7 | TGCTATCCTGCCCTCTGTCT | GCAAGGAAACCAGCACCATG |
| Atg12 | AAGTGGGCAGTAGAGCGAAC | CACGCCTGAGACTTGCAGTA |

All these information has also been mentioned in our earlier papers **[13, 19].**
