# Supplementary figures and images for: Desialylation of Atg5 by sialidase (Neu2) enhances autophagosome formation to induce anchorage-dependent cell death in ovarian cancer cells
Source: Cell Death Discov. 2021 Feb 1;7:26. doi: 10.1038/s41420-020-00391-y (PMC7851153; doi:10.1038/s41420-020-00391-y)

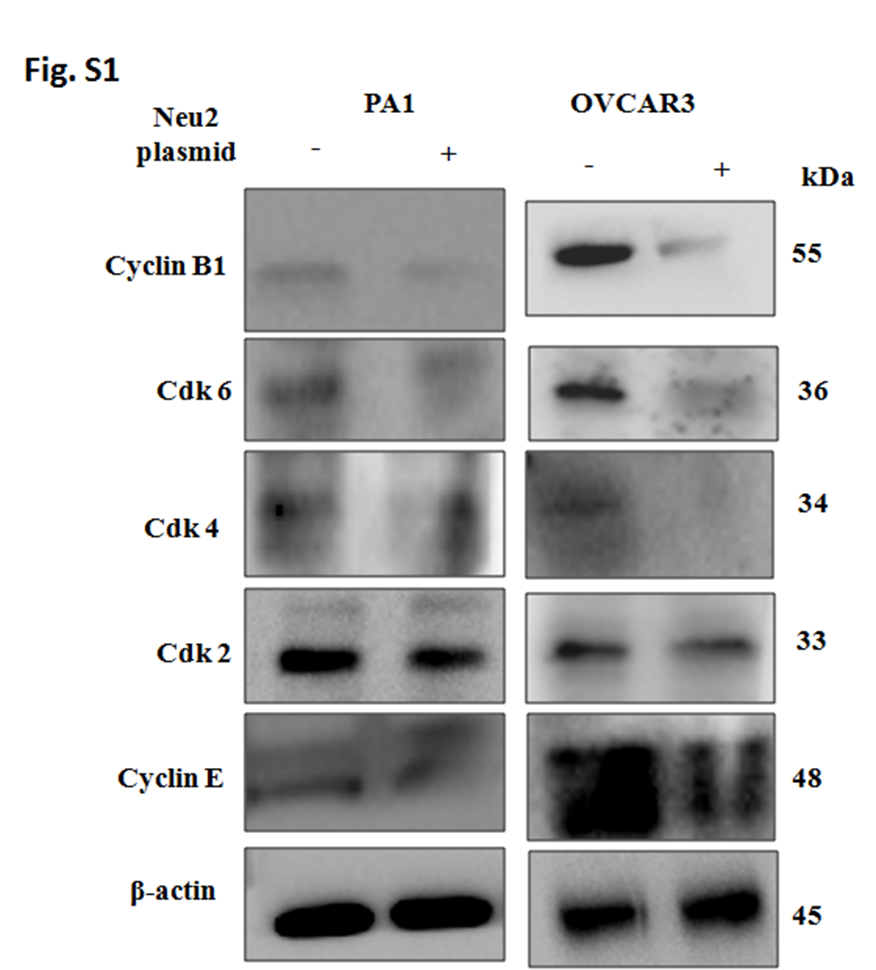

Supplement: Supplementary file 4 — Figure S1 Overexpressed Neu2 halts the cell cycle in ovarian cancer cells. [file 41420_2020_391_MOESM4_ESM.tif]

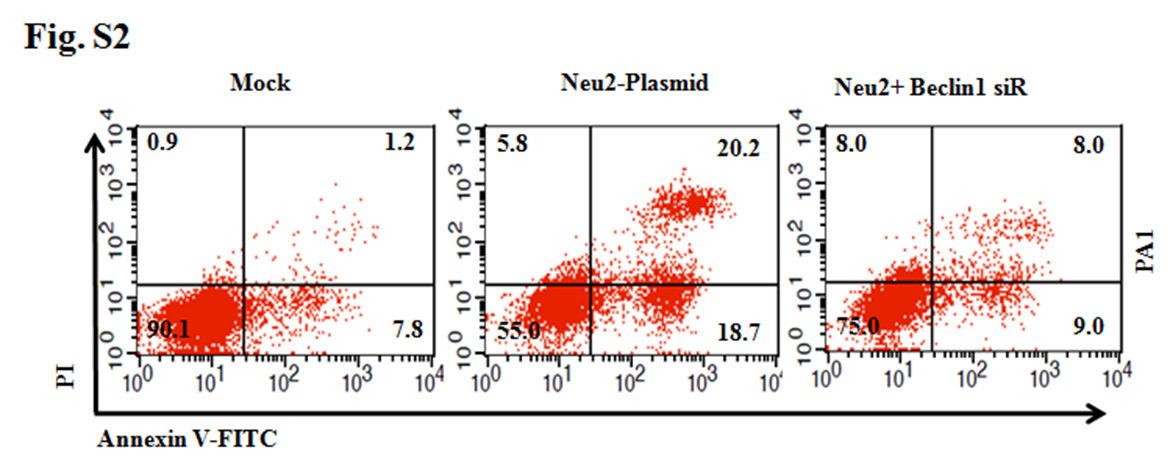

Supplement: Supplementary file 5 — Figure S2 Beclin-1 knockdown reduced the Neu2-induced apoptosis in ovarian cancer cells. [file 41420_2020_391_MOESM5_ESM.tif]
